# Torque Loss, Survival, and Strain Distribution of Implant-Supported Prostheses with Zirconia and Cobalt–Chromium Hybrid Abutments

**DOI:** 10.3390/medicina61020274

**Published:** 2025-02-05

**Authors:** Renata Cristina Silveira Rodrigues, Lívia Fiorin, Adriana Cláudia Lapria Faria, Estevam Augusto Bonfante, Ricardo Faria Ribeiro

**Affiliations:** 1 Department of Dental Materials and Prosthesis, School of Dentistry of Ribeirao Preto, University of Sao Paulo (USP), Ribeirao Preto 14040-904, SP, Brazil; liviafiorin01@gmail.com (L.F.); adriclalf@forp.usp.br (A.C.L.F.); 2 Department of Prosthodontics and Periodontology, Bauru School of Dentistry, University of Sao Paulo (USP), Bauru 17012-901, SP, Brazil; estevam.bonfante@fob.usp.br

**Keywords:** dental implant/abutment design, dental implant, single tooth, zirconium, cobalt–chromium alloys, titanium, fatigue, step-stress accelerated life testing, survival, reliability, materials testing, stress, mechanical

## Abstract

*Background and Objectives*: The manufacturing of single crowns using hybrid abutments is an alternative that may be interesting in clinical practice, combining the advantages of the different materials used in a personalized design for each case. The purpose of this in vitro study was to evaluate the torque loss, survival, reliability, failure mode, and strain distribution of implant-supported prostheses with zirconia (Zir) and cobalt–chromium (Co-Cr) hybrid abutments. *Materials and Methods*: Abutments were milled by CAD/CAM and divided into two groups according to the materials used, Zir and Co-Cr, and cemented on titanium bases screwed to dental implants. Monolithic zirconia crowns were cemented on the abutments. The implant/abutment/crown sets were subjected to thermomechanical cycling (*n* = 10) (2 Hz, 140 N, 1 × 10^6^ cycles, immersed in water at 5–55 °C) to evaluate the torque loss. The single load to fracture test (SLF) was performed to design the loading profiles (light, moderate, and aggressive) of the step-stress accelerated life testing (SSALT) (*n* = 21) to evaluate the survival and reliability. The representative fractured specimens were analyzed under optical and scanning electron microscopy. The digital image correlation (DIC) (*n* = 1) was performed using specimens embedded in polyurethane resin models that received static point loading, and the strain distribution was analyzed. *Results*: There was no difference in torque loss, survival, or reliability between zirconia and Co-Cr abutments. An analysis of the fractured surfaces showed that the abutments presented the same failure mode, where the fracture probably started in the titanium base/screw. The zirconia abutment model presented only compressive strains around the implant, while the Co-Cr abutment model showed tensile and compressive strains in the middle of the implant; however, all strains were within the clinically acceptable limits. There was a strain concentration in the titanium base close to the implant platform for both groups. *Conclusions*: Zirconia and Co-Cr hybrid abutments presented similar torque loss, survival, reliability, and failure modes, but the abutment material influenced the strain distribution around the implant. The titanium base screw was the weakest link in the system.

## 1. Introduction

Implant-supported restorations are a widely accepted treatment modality for the replacement of missing teeth [1,2]. The selection of implant abutment and abutment material is fundamental for the long-term success of implant-supported restorations because it plays a critical role in supporting the prosthesis and strain distribution of the material to bone tissue [3,4]. The most used abutments for cement-retained crowns are titanium prefabricated and hybrid abutments. Titanium prefabricated abutments have been preferred for many years due to their superior mechanical properties; however, zirconia abutments provide a better esthetic outcome, especially in patients with a thin gingival profile [5,6,7]. Initially, a one-piece zirconia abutment was fabricated, resulting in implant wear and deformation, and leading zirconia to fracture [8]. Two-piece or hybrid abutments were introduced to solve this problem, which include a titanium base screwed into the implant and the abutment cemented to the titanium base [6,9]. The manufacturing of single crowns using hybrid abutments is an alternative that may be interesting in clinical practice, combining the advantages of the different materials used in a personalized design for each case.

Computer-aided design and computer-aided manufacturing (CAD/CAM) technology has been used to manufacture abutments, involving fewer manual steps, allowing a simplified manufacturing process associated with high predictability [10,11]. Zirconia, lithium disilicate, polyetheretherketone (PEEK), and recently cobalt–chromium (Co-Cr) are available in blocks to mill hybrid abutments [9]. Initially, the Co-Cr alloy was predominantly utilized for the construction of metallic frameworks for partial removable prostheses; however, the application has expanded to abutments. The principal advantages contributing to the growing use of Co-Cr is its cost-effectiveness [12] and the possibility of milling high machinable presintered metal blocks. The conventional casting for Co-Cr requires higher melting temperatures than golden alloys, increasing contraction during cooling [13], while CAD/CAM technology allows the obtaining of more precision prosthetic pieces [11,14].

However, there remain several uncertainties surrounding the use of hybrid abutments. Issues such as fractures of the titanium base and complications related to the cementation of zirconia abutments have been reported [15,16,17,18,19]. Co-Cr hybrid abutments have been assessed for marginal fit [20,21,22], bacterial adhesion [22], torque loss [23], and compressive strength under static load [23], but their survival probability has not been thoroughly investigated. Another factor for which information is lacking is the behavior of the association of different materials in the oral environment regarding corrosion resistance and possible ion release. Since implant-supported restorations are exposed to cyclic loading during repetitive function, failures often arise over time. Step-stress accelerated life testing can simulate clinical failure modes in vitro, making it a useful method for predicting the survival probability of implant-supported restorations [24,25].

The study of the mechanical behavior of implant-supported restorations using hybrid abutments can provide important information for clinicians and researchers, offering elements that can help manufacturers with future designs that allow the safe use of these components. Therefore, the purpose of this in vitro study was to evaluate the torque loss, survival, reliability, failure mode, and strain distribution of implant-supported restorations with zirconia and Co-Cr hybrid abutments obtained by CAD/CAM. The null hypothesis was that the materials (zirconia or Co-Cr) used to manufacture hybrid abutments did not influence the torque loss, survival, reliability, failure mode, or strain distribution of the implant-supported restorations.

## 2. Materials and Methods

The dental implant with internal conical connection (5 × 13 mm, Singular Implants, Parnamirim, Brazil) were placed in a dental mannequin, the scan body (Singular Implants) was positioned, and the scan (Ceramill Map400, AmannGirrbach, Mäder, Austria) was performed. The abutments were designed (Ceramill M-Plant, AmannGirrbach Mäder, Austria) and milled (Ceramill Motion 2, AmannGirrbach, Mäder, Austria) in presintered zirconia (Ceramill ZI, AmannGirrbach, Koblach, Austria) and presintered Co-Cr (Ceramill Sintron, AmannGirrbach, Koblach, Austria) blocks by CAD/CAM. The abutments were sintered in a furnace (In Fire HTC Speed, Dentsply Sirona, Bensheim, Germany) according to the manufacturer’s instructions, with argon atmosphere for Ceramill Sintron. The dental implants were embedded in polyurethane resin (F160, Axson, Saint-Ouen-l’Aumône, France). The titanium bases with a 2.5 mm strap (TiBase, Singular Implants, Parnamirim, Brazil) were screwed to the implants with the torque recommended by the manufacturer (32 Ncm) by using a digital torque gauge (TQ 680, Instrutherm, São Paulo, Brazil). The abutments were subjected to sandblasting with 50 μm of aluminum oxide (Basic Classic, Renfert, Hilzingen, Germany) under a pressure of 1.5 bar, 8 sec and 25 mm distance using a special device made to support the sample. After, the abutments were cemented to the titanium bases using a dual resin cement (Megalink, Odontomega, Ribeirão Preto, Brazil). Excess cement was removed, and photopolymerization was performed on all faces for 20 s each (Valo, Ultradent, South Jordan, UT, USA) and kept under a weight of 439 g using a modified surveyor for 5 min. Monolithic zirconia crowns were designed on these abutments, milled with presintered zirconia blocks (Ceramill Zolid, AmannGirbach, Koblach, Austria) by CAD/CAM, and sintered in a furnace (InFire HTC Speed, Dentsply Sirona, Bensheim, Germany). For the thermomechanical cycling test, the monolithic zirconia crowns were fixed to the abutments with polyether (Impregun soft, 3M ESPE, Seefeld, Germany), also using a modified surveyor to standardize the positioning, and for the step-stress accelerated life testing (SSALT) and the digital image correlation (DIC), they were subjected to sandblasting with aluminum oxide (Basic Classic, Renfert, Hilzingen, Germany) and cemented to the abutments using a dual resin cement (Megalink, Odontomega, Ribeirão Preto, Brazil) using the same procedures described above for the cementation of abutments to titanium bases. The implant/abutment/crown sets were divided into two groups according to the material used to manufacture abutments: zirconia and Co-Cr.

The torque loss was calculated before (initial torque loss) and after thermomechanical cycling (post-load torque loss). To evaluate the initial torque loss, the abutments were installed with torque recommended by the manufacturer (initial torque). After 10 min, they were unscrewed (preload removal torque) to evaluate the settling effect. Then, they were retorqued, and 10 min later, torque was confirmed (confirmation torque). Next, the implant/abutment/crown sets (*n* = 10) [3] were positioned in a 30° inclination matrix, according to the ISO 14801:2016 [26] and subjected to thermomechanical cycling applying a static load of 140 N to the palatine concavity of the crown with 2 Hz of frequency until completing 1 × 10^6^ mechanical cycles [27,28] and 2002 thermal cycles, with each thermal cycle including the immersion in water at 5 and 55° C. After thermomechanical cycling, the removal torque was measured (post-load removal torque). The percentage of torque loss before and after thermomechanical cycling was calculated based on the following formulas [27]:

$$Initial\;torque\;\left(\%\right)=\frac{Initital\;torque-preload\;\;removal\;torque}{Initial\;\;torque}\times 100$$

(1) $$Post\;load\;torque\;loss\;\left(\%\right)=\frac{Confirmation\;torque-Post\;load\;removal\;torque}{Confirmation\;torque}\times 100$$

**Figure 1:**
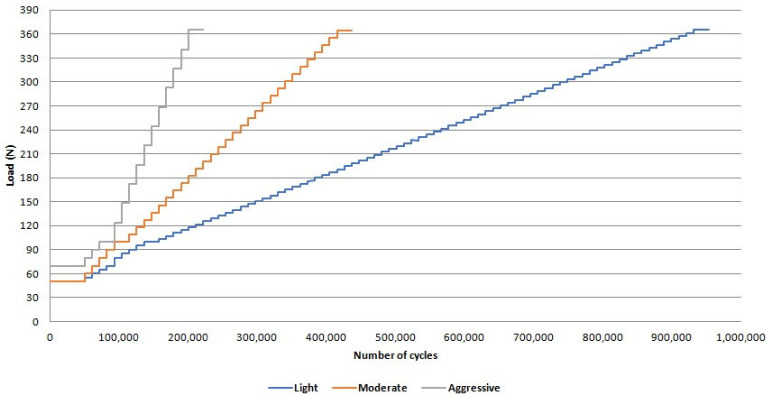
Load and number of cycles of the loading profiles of the step-stress accelerated life testing.

The survival and reliability of implant/abutment/crown sets were determined by the SSALT (*n* = 21). For this, the implant/abutment/crown sets were positioned in a 30º inclination matrix [3], and the single load to fracture test (SLF) (*n* = 3) was performed in a universal testing machine (Universal Testing Machine, Biopdi, São Carlos, Brazil), applying a compressive load using a 1000 kgF load cell with a displacement of 1 mm/min until fracture. The load of light (*n* = 9), moderate (*n* = 6), and aggressive (*n* = 3) loading profiles of SSALT ranged between 20% and 60% of the SLF mean value [24,25] (Figure 1). The fatigue testing equipment (Biocycle, Biopdi, São Carlos, Brazil) was used with an isometric loading protocol and 4 Hz frequency. During the test, the implant/abutment/crown sets were positioned in the same matrix of SLF, the load was applied to the palatine concavity of the monolithic crown, and they were subjected to thermomechanical cycling immersed in water at 5 and 55 °C until the limit of cycles or failure [27,28]. At the end of each step, the specimens were analyzed to verify the presence of deformations and/or fractures. It was considered a failure when there was a fracture in the implant, the titanium base, or the abutment/crown set.

After SSALT, the implant/abutment/crown sets were analyzed under optical and scanning electron microscopy. The images were captured with a digital camera (DFC 250, Leica) and analyzed with a stereoscopic magnifying glass (S8APO, Leica Microsystems, Taiwan). A representative fractured specimen of each group was selected, cleaned in an ultrasonic tank for 3 min immersed in ethanol, positioned on a metal stub, and metallized with a gold–palladium alloy (Sputter Coater, Cressington, Ted Pella, Redding, Canada). Next, it was qualitatively analyzed by scanning electron microscopy (SEM) using a microscope (EVO-MA10, Zeiss, Oberkochen, Germany). The images were taken using 80× and 500× magnification.

The strain distribution around the zirconia and Co-Cr abutments was analyzed by DIC. For this, the master model of polymethylmethacrylate (PMMA) was made with dimensions of 55 × 30 × 14 mm (length, height, and depth, respectively), and the implant/abutment/crown set was fixed with cyanoacrylate glue (Super Bonder, Loctite, São Paulo, Brazil). After 24 h, an impression of the PMMA master model with silicone (Silikon, Odontomega, Ribeirão Preto, Brazil) was made, and the implant/abutment/crown set was embedded in polyurethane resin (F16, Axson, Saint-Ouen-l’Aumône, France) to capture the implant position. Next, the model surface and implant/abutment/crown set were painted with a thin layer of white paint (Colorgin Premium, Sumaré, Brazil) and small black spray dots (Colorgin Premium). The DIC complete system (StrainMaster, GmbH, Germany) included two charge-coupled device digital cameras (Image E-lite 2M, 1101132, LaVision GmbH), with a resolution of 1039 × 1395 pixels, which was used to capture the images of the model under loading, and software (Davis 8.0, LaVision GmbH, Göttingen, Germany) for image analysis and strain calculation. Two conditions were analyzed: rehabilitation with a zirconia abutment and crown and rehabilitation with a Co-Cr abutment and zirconia crown.

The strain distribution around the implant was evaluated by the application of 250 N, while the strain distribution in the crown, abutment, and titanium base was evaluated using 300 N. Static load was applied by the universal testing machine (Biopdi, São Carlos, Brazil) using a 50 kgF load cell with a displacement of 1 mm/min. During load application, point dislodgement was tracked by the software to calculate the strains on the model surface and in the crown, abutment, and titanium base. Three loadings were performed on each model to verify the repeatability and reliability of the DIC. The qualitative analysis of the images obtained by DIC was based on a color scale, where positive values (from yellow to red) referred to tensile strains and negative values (from green to blue) to compressive strains.

The results of torque loss were analyzed by the software SPSS (v20.0.0, SPSS Inc, IBM SPSS, New York, NY, USA) with a linear regression model with random effects. For the SSALT, the use level probability Weibull curve (probability of failure versus number of cycles) was calculated (Alta Pro 9, ReliaSoft, Tucson, AZ, USA) using, as a parameter, 60% of the maximum load found in SLF and a bilateral 90% confidence interval (CI). Reliability was calculated for a mission of 200,000 and 300,000 cycles at 200 N and 300 N, and differences between missions were identified using the Weibull calculation with a two-way 90% confidence interval.

## 3. Results

The results of torque loss are shown in Table 1. There was no difference between torque loss comparing zirconia and Co-Cr abutments before (*p* = 0.386) and after (*p* = 0.865) thermomechanical cycling. Comparing the initial and final times within the same group, it was observed that there was a torque loss for both groups: zirconia (*p* = 0.018) and Co-Cr (*p* = 0.012) (Table 1).

The mean values obtained in SLF (N) were zirconia = 555.37 and Co-Cr = 380.75. The mean of the two groups (468.06 N) was used to establish the applied load on the SSALT light, moderate, and aggressive loading profiles. The β mean values derived from the use level probability Weibull (90% two-way CI) were 1.91 and 3.51 for zirconia and Co-Cr, respectively (Table 2), indicating that fatigue influenced the survival of implant/abutment/crown sets, with the accumulation of damage being the main factor for failure.

Table 3 and Table 4 shows the reliability of zirconia and Co-Cr abutments. There was no difference between groups for the reliability of the missions of 200 N and 300 N. However, it was possible to observe that the reliability of both groups drops significantly in the mission with 300,000 cycles at 300 N, where 46% of failures were observed for zirconia and 48% were observed for Co-Cr.

**Figure 2:**
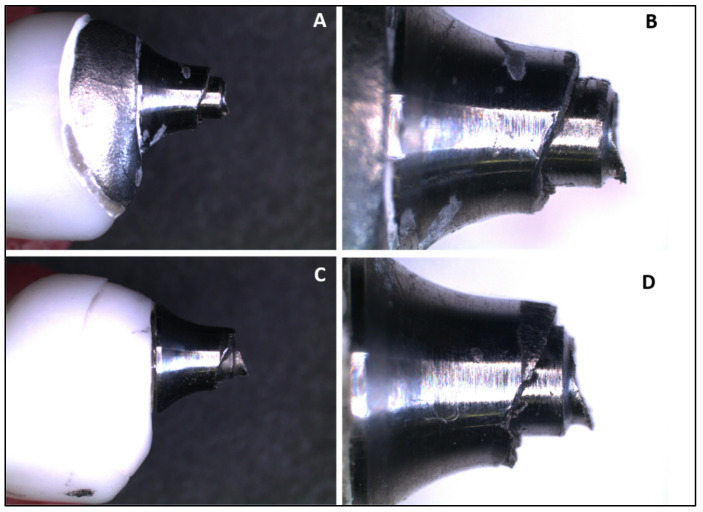
(**A**) Fracture of the Co-Cr abutment screw, 10× magnification; (**B**) fracture of the Co-Cr abutment screw, 25× magnification; (**C**) fracture of the zirconia abutment screw, 10× magnification; (**D**) fracture of the zirconia abutment screw, 25× magnification.

**Figure 3:**
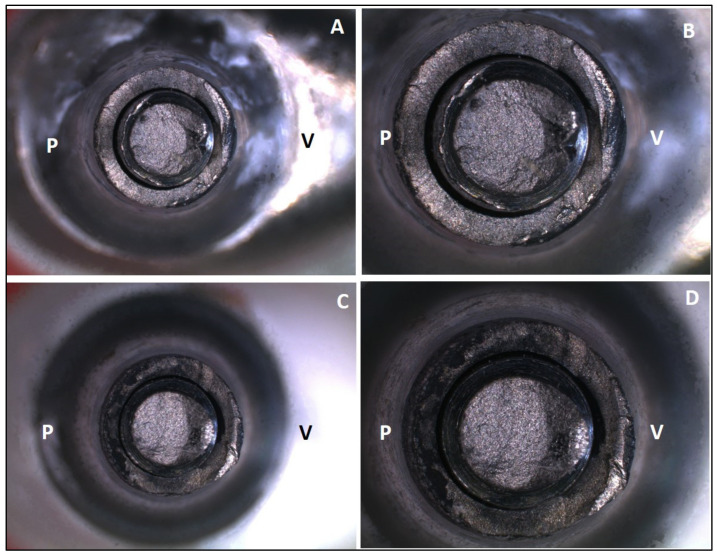
(**A**) Internal region of the Co-Cr abutment screw, 10× magnification; (**B**) internal region of the Co-Cr abutment screw, 32× magnification; (**C**) internal region of the zirconia abutment screw, 10× magnification; (**D**) internal region of the zirconia abutment screw, 32× magnification. V = vestibular surface and P = palatal surface.

**Figure 4:**
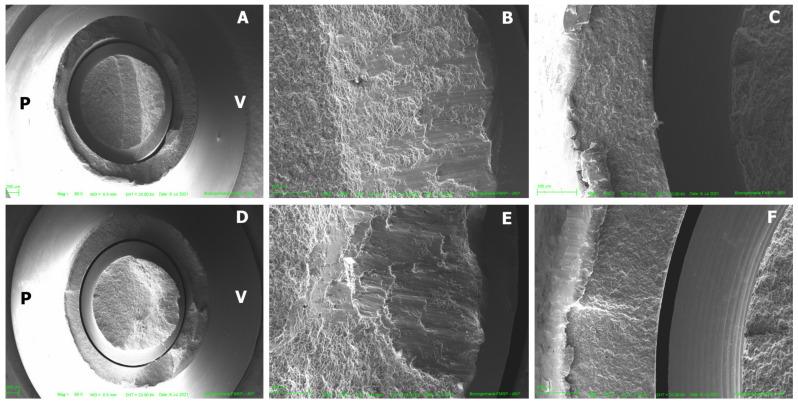
Scanning electron microscopy. (**A**) Fractured region of the Co-Cr abutment, 80× magnification; (**B**) Co-Cr abutment in the vestibular region, 500× magnification; (**C**) Co-Cr abutment in the palatal region, 500× magnification; (**D**) fractured region of the zirconia abutment, 80× magnification; (**E**) zirconia abutment in the vestibular region, 500× magnification; (**F**) zirconia abutment in the palatal region at 500× magnification. V = vestibular surface and P = palatal surface.

Figure 2 and Figure 3 illustrate the failure mode after SSALT by optical microscopy. The region of fracture was similar in both groups and corresponds to the region where the threads begin (Figure 2). The fracture occurred in the region of the screw corresponding to the palatal surface of the monolithic crown, where the tensile forces were concentrated during SSALT (Figure 3). Figure 4 illustrates the failure mode after SSALT by SEM. In the region corresponding to the buccal face (Figure 4B,E), it was possible to observe compression areas in the region of the abutment screw. While in the region corresponding to the palatal face (Figure 4C,F), it was possible to observe tensile areas and the beginning of the fracture of the screw.

**Figure 5:**
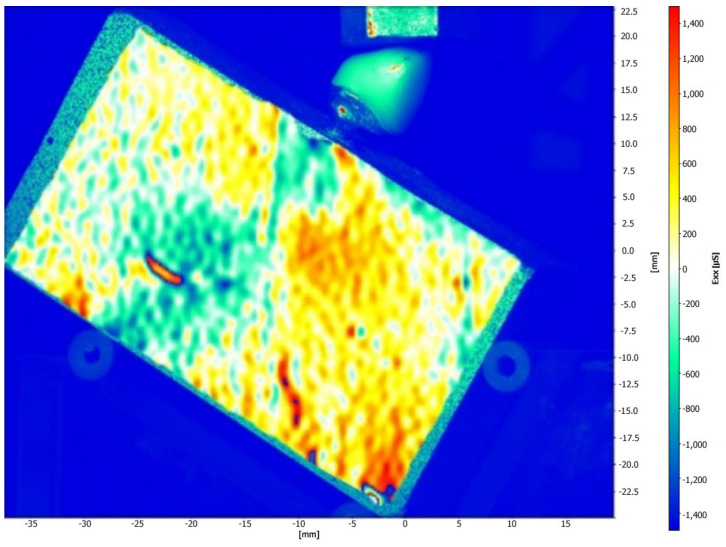
Strain distribution around the implant after the load was applied on the implant/abutment/crown set using Co-Cr abutments.

**Figure 6:**
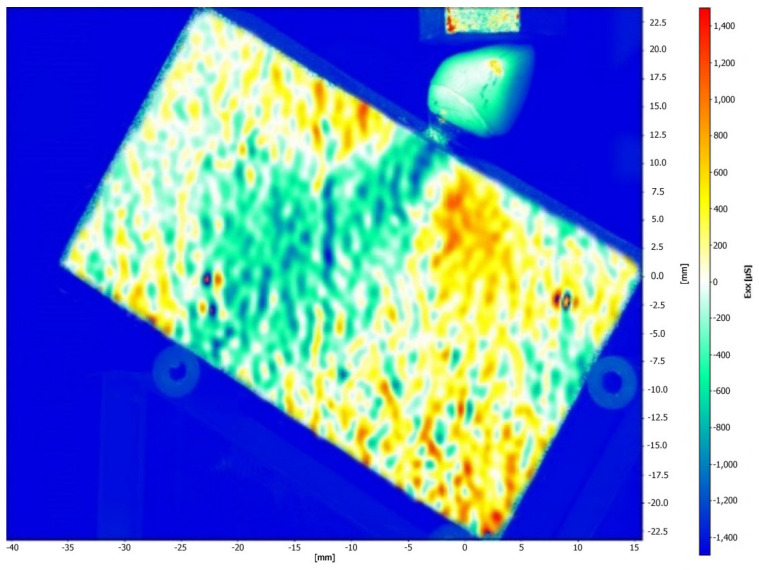
Strain distribution around the implant after the load was applied on the implant/abutment/crown set using zirconia abutments.

**Figure 7:**
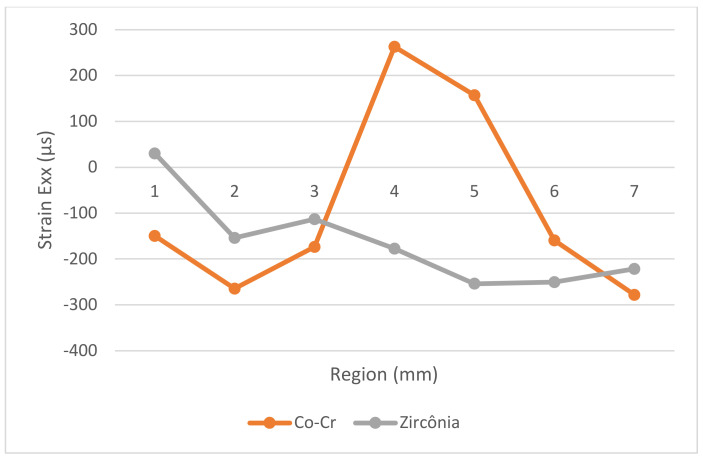
Strain distribution (µS) around the implant (mm) after the load was applied on the implant/abutment/crown set for the Co-Cr and zirconia groups.

**Figure 8:**
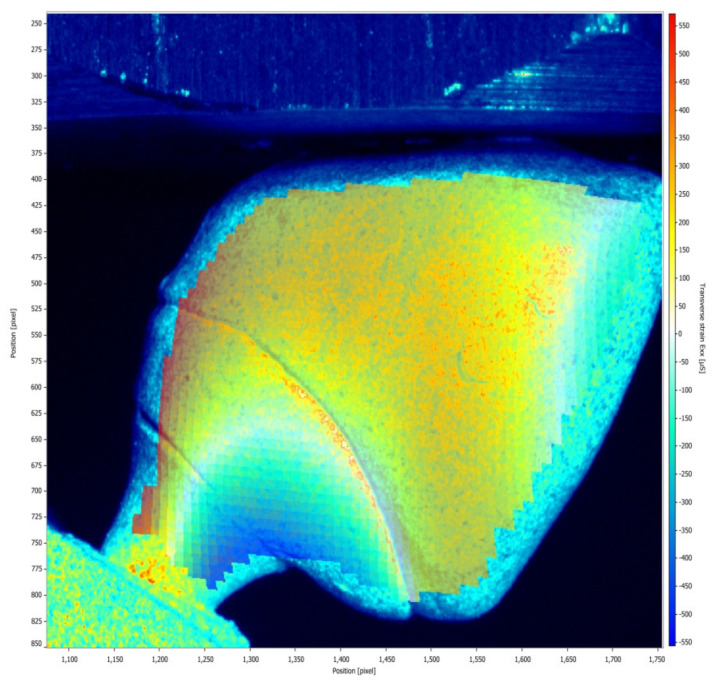
Strain distribution in the abutment/crown after the load was applied on the set for the Co-Cr group.

**Figure 9:**
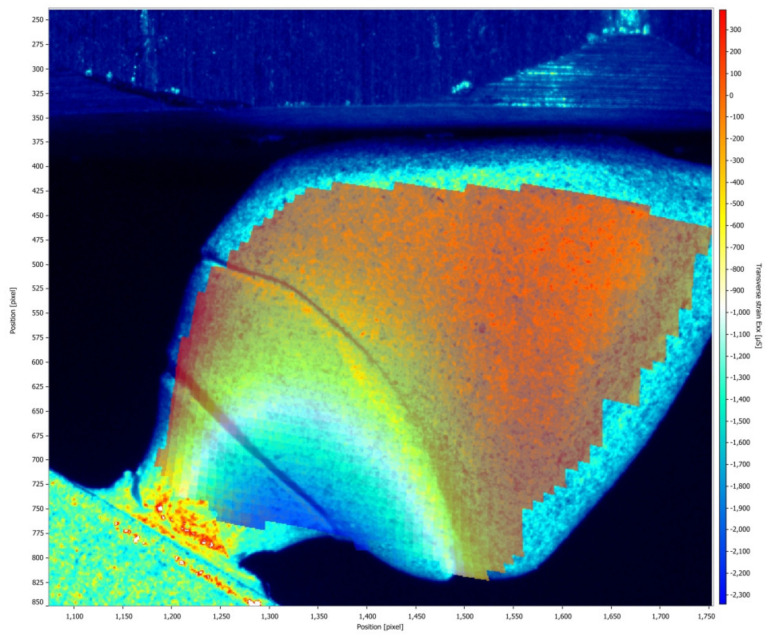
Strain distribution in the abutment/crown after the load was applied on the set for the zirconia group.

**Figure 10:**
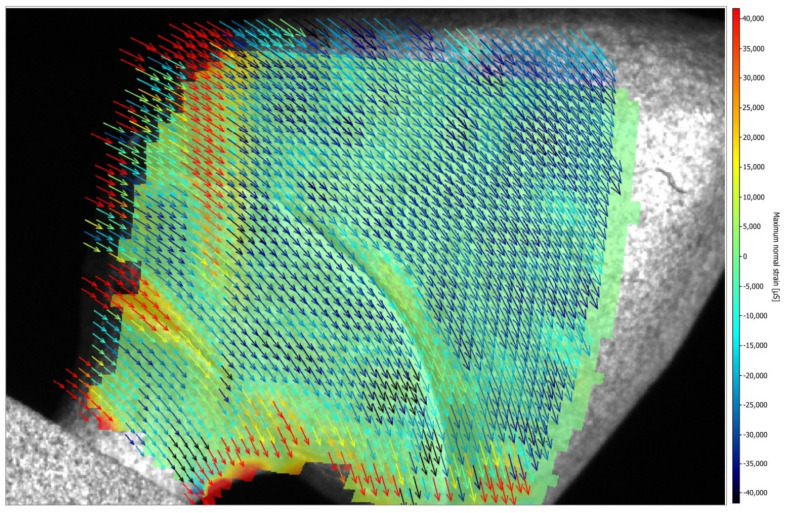
Vectorization of the maximum principal strains (µs) on the set.

**Figure 11:**
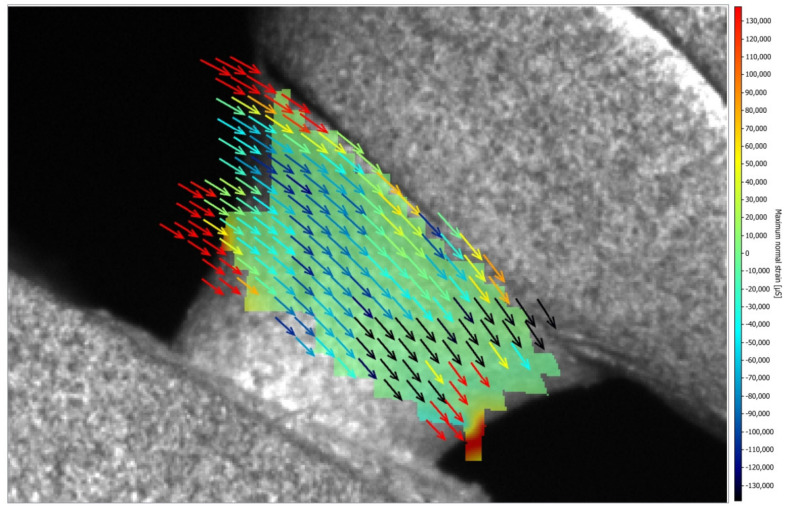
Vectorization of maximum principal strains (µs) on the titanium base.

Figure 5 and Figure 6 illustrate the strain distribution around the implant after the load was applied. The Co-Cr abutments presented tensile (colors varying from yellow to red) and compressive strains in the middle of the implant region (Figure 5), while zirconia abutment presented a predominance of compressive strains (colors ranging from green to blue) around the implant (Figure 6). Figure 7 showed that zirconia abutments presented a predominance of compressive strains with a maximum value of −254 µs, while Co-Cr abutments presented a maximum value of −272.7 µs for compressive strains, and a maximum value of 278 µs for tensile strains. DIC also revealed the predominance of tensile strains in the region of load application (palatine concavity of the monolithic crown) and in the titanium base region (Figure 8 and Figure 9). Figure 10 illustrated the vectorization of the maximum principal strains on the set, while Figure 11 showed the titanium base.

## 4. Discussion

The null hypothesis was partially accepted because the materials (zirconia and Co-Cr) used to manufacture hybrid abutments did not influence torque loss, survival, reliability, and failure mode, but they influenced the strain distribution on the implant/abutment/crown sets. The torque loss of zirconia and Co-Cr abutments was similar after thermomechanical cycling. This result was in accordance with previously conducted studies that did not find differences among torque losses of different abutments [3,29]. Elsayed et al. [2] explained that the torque loss was caused by micromovements during thermomechanical cycling. This result showed that the coronal portion of the abutment manufactured with zirconia and Co-Cr had no impact on the behavior of the titanium base in the face of these micromovements, as both materials showed similar behavior. When the torque loss of each group was compared before and after thermomechanical cycling, both showed a significant torque loss, meaning that the post-thermomechanical cycling torque was lower than the initial torque. It is important to emphasize that during thermomechanical cycling, no axial loads were applied except for loads at 30° on the monolithic crown that increased the lever arm, leading to a higher movement of the set.

The implant/abutment/crown sets were subjected to the SSALT to evaluate the survival and reliability of hybrid abutments. The results showed that there was no difference between the zirconia and Co-Cr hybrid abutments to the analyzed missions. The Weibull analyses showed that the failures occurred due to damage accumulation, presenting β values higher than 1. Similar results were observed by Barbosa-Júnior et al. [30] that found no difference in the survival of zirconia and PEEK hybrid abutments after SSALT. This analysis also showed similar reliability for both abutments, with values around 90% for the mission with 200 N of load and 200,000 and 300,000 cycles, respectively. For a mission of 300 N and 200,000 cycles, the reliability remained high (76% for zirconia and 85% for Co-Cr), but with the increase in the number of cycles to 300,000, this reliability dropped to 54% for zirconia and 52% for Co-Cr. SSALT showed that these hybrid abutments have good reliability for clinical use because most fractures occurred with loads around 300 N and after numerous cycles. It is noteworthy that the frequency used in the SSALT was 4 Hz and that the load was applied at 30°, making the test quite demanding on the implant/abutment/crown set. The chewing loads in the anterior region range from 129.5 N to 226.6 N, and patients with parafunctional habits average 796.8 N [31,32]. Therefore, the hybrid abutments tested can be indicated for the rehabilitation of the anterior region, since the first failures of the abutments occurred with loads close to 220 N, being 180 N for the zirconia abutments and 176.2 N for the Co-Cr abutments. Patients with parafunctional habits must be carefully evaluated, and rehabilitation with hybrid abutments may not be the best choice.

After SSALT, the failure mode of the implant/abutment/crown sets was evaluated, and fractures occurred in a similar location for both groups. The fracture occurred in the titanium base, where the fracture of the screw and the base of the abutment can be observed. Optical microscopy and SEM image analysis suggested that the fracture started at the screw, in the region of the first threads, probably originating on the side facing the palatal concavity of the monolithic crown, where the load was applied during SSALT. The possible micromovements observed from the beginning of the fracture probably determined the fracture of the screw, and then the complete fracture of the titanium base occurred. Similar results were found by Silva et al. [16], who reported that most of the failures after SSALT occurred in the screw and led to abutment fracture. They highlighted that these failure modes indicate that the abutment design transfers significant strains to the screw, leading to fracture. Barbosa Júnior et al. [30] reported that failure mode is more related to the crown material than to the hybrid abutment material. These authors analyzed the failures of zirconia abutments restored with monolithic zirconia crowns, and most of the failures occurred in the abutment screw. According to the authors, the higher structural strength of the monolithic zirconia crowns allowed them to resist the strains generated by the application of load and transferred to the screw. The fracture of the ceramic and problems with adhesion between crown and abutments were reported by other studies [3,17,19]; however, in this study no fracture was observed in the ceramic, and no adhesion failure occurred on the monolithic crown, in agreement with Yilmaz et al. [29]. The zirconia and Co-Cr hybrid abutments manufactured by CAD/CAM remained perfect, indicating that the weakest link was the titanium base/screw set.

The DIC revealed that zirconia abutments generated compression strains in the implant region. The Co-Cr abutments generated compression and tensile strains, which are more evident in the middle of the implant, approximately 4 and 5 mm from the cervical region. The interpretation of these data considers that bone physiology presents a reaction to the transmission and concentration of strains arising from occlusal loading. Bone tissue has lower resistance to tensile strains, while compressive strains push the bodies against each other and tend to maintain the integrity of the implant interface, while tensile strains tend to rupture this interface. The data are important as they show that the cortical bone tissue undergoes a rupture of the bone–implant interface when exposed to tensile strains, making them more destructive in the biomechanical analysis [33]. The values obtained in this study are within acceptable standards and are not harmful to bone tissue, as indicated in a previous study [34]. According to Frost [34], strengths between 200 μs and 400 μs do not cause damage to bone density, and values between 4000 μs and 25,000 μs can cause microscopic damage to bone tissue. Concordant data were observed by Duyck et al. [35], who suggested that the value for starting bone resorption is 4200 μs. In the present study, the maximum compression strain values found were −254 µs for the zirconia abutment and −272.7 µs for the Co-Cr abutment, and the maximum tensile strain value was 278 µs for the Co-Cr abutment. These values were all within low limits that would not cause damage to the bone tissue.

The DIC also revealed the presence of higher tensile strain in the palatal region of the monolithic crown, with high intensity for the zirconia abutment. Both groups presented strain concentration in the abutment, in the region where the fracture occurred during function. Clinically, when a fracture occurs, the removal of the fractured screw inside the implant is quite difficult and can damage the internal portion of the implant, making the subsequent use of the implant unfeasible.

The present study showed that the implant/abutment/crown set with zirconia and Co-Cr hybrid abutments presented similar torque loss, survival, reliability, and failure modes, but differences on the strain distribution. The zirconia abutments caused a predominance of compressive strains around the implant, while the Co-Cr abutments caused tensile and compressive strains in the middle of the implant. There was a concentration of tensile strains in the titanium base of both abutments, which probably caused the titanium base fracture.

This study has limitations, including that thermomechanical cycling and SSALT did not assess complex factors found in the oral environment, such as occlusal loading dynamics, neuromuscular forces, and parafunctional habits; the models used for DIC were manufactured using a polyurethane resin that was solid, homogeneous, without porosity, and isotropic. Despite these limitations, the results of this in vitro study demonstrated that the titanium base/screw was the most fragile part of the set, and the material used on the abutment had little influence on the mechanical behavior of the implant/abutment/crown set, forming the basis for future research.

There are several other aspects that need to be investigated for the use of hybrid abutments and that were not the subject of this study. For example, it is necessary to investigate the corrosion resistance and possible ion release from the hybrid abutments into the oral environment, considering the association of different materials in the sets.

Also, modifications to the design of the titanium base are necessary to strengthen its internal position and potentially improve its resistance, allowing for even safer clinical use. Therefore, studies on this aspect are also necessary, generating more complete information so that professionals can select their options more consciously.

## 5. Conclusions

Based on the results of the present study and within the limitations of the methods used, the following can be concluded:

- There was no difference between torque loss of zirconia and Co-Cr abutments after the thermomechanical cycling;
- There was no difference between survival and reliability of zirconia and Co-Cr abutments for the evaluated missions after the step-stress accelerated life testing;
- Failure mode was similar for zirconia and Co-Cr hybrid abutments, and fracture occurred in the titanium base ;
- The model of zirconia abutment presented only compression strain, while the model of Co-Cr abutment presented compression and tensile strains; however, all strains are within acceptable limits, indicating the safe use of both types of hybrid abutments.

## Figures and Tables

**Table 1:** Torque loss before and after thermomechanical cycling.

Groups	Thermomechanical Cycling	Mean	Standard Deviation	Lower Limit	Upper Limit
Co-Cr	Before	22.18 ^Aa^	7.43	11.30	35.00
	After	42.75 ^Ab^	24.57	−17.30	66.90
Zirconia	Before	18.75 ^Aa^	9.70	6.20	32.20
	After	40.71 ^Ab^	28.20	−6.80	78.20

^A, B^ Different uppercase letters indicate statistical difference (*p* < 0.05) between groups, and ^a, b^ different lowercase letters indicate statistical difference (*p* < 0.05) between initial and final.

**Table 2:** Weibull modulus.

	Groups	
	Co-Cr	Zirconia
Upper limit	5.56	3.54
β	3.51	1.91
Lower limit	2.21	1.03

**Table 3:** Reliability for missions with 200,000 and 300,000 cycles at 200 N.

Missions	Groups	Upper Limit	Reliability 200 N	Lower Limit
200,000 cycles	Co-Cr	99	96	81
	Zirconia	97	91	71
300,000 cycles	Co-Cr	93	83	62
	Zirconia	91	81	61

**Table 4:** Reliability for missions with 200,000 and 300,000 cycles at 300 N.

Missions	Groups	Upper Limit	Reliability 300 N	Lower Limit
200,000 cycles	Co-Cr	98	85	31
	Zirconia	95	76	19
300,000 cycles	Co-Cr	87	52	5
	Zirconia	86	54	9

## Data Availability

Data supporting the results of this study are available in the article and can be requested from the corresponding authors.
